# Complete plastome sequence of *Mallotus peltatus* (Geiseler) Müll. Arg. (Euphorbiaceae): A beverage and medicinal plant in Hainan, China

**DOI:** 10.1080/23802359.2020.1719935

**Published:** 2020-01-31

**Authors:** Xiu-Rong Ke, Xiao-Feng Zhang, Hong-Xin Wang, Zhi-Xin Zhu, Juan-Ling Li, Hua-Feng Wang

**Affiliations:** a Key Laboratory of Tropical Biological Resources of Ministry of Education, School of Life and Pharmaceutical Sciences, Hainan University, Haikou, China;; b College of Forestry, Hainan University, Haikou, China

**Keywords:** *Mallotus peltatus*, plastome, phylogeny, genome structure, Euphorbiaceae

## Abstract

*Mallotus peltatus* is a tropical plant of the Euphorbiaceae family, which could be used as a beverage and medicine in Hainan, China. Here, we report and characterize the complete plastome of *M. peltatus.* The complete plastome is 163,304 bp in length and contains a typical structure and gene content of angiosperm plastome, including two inverted repeat (IR) regions of 27,112 bp, a large single-copy (LSC) region of 89,886 bp and a small single-copy (SSC) region of 18,840 bp. The plastome contains 131 genes, consisting of 78 unique protein-coding genes, 30 unique tRNA gene, four unique rRNA genes (5S rRNA, 4.5S rRNA, 23S rRNA and 16S rRNA), and eight pseudogenes. The overall A/T content in the plastome of *M. peltatus* is 64.02%. The complete plastome sequence of *M. peltatus* will provide a useful resource for the conservation genetics of this species as well as for phylogenetic studies in Euphorbiaceae.

## Introduction

*Mallotus peltatus* (Geiseler) Müll.Arg. (Euphorbiaceae) is an evergreen shrub or small tree ranging from two to eight m tall (Li et al. 2008). *M. peltatus* is a native plant of Hainan Island in China, which is used as a beverage and medicine for local people (Yan et al. 2019). At present, the complete plastome information and systematic position of *M. peltatus* has been rarely studied and reported. Hence, the genetic and genomic information is essential needed to aid to its resource exploitation and conservation. Here, we report and characterize the complete plastome of *M. peltatus* (GenBank accession number: MN885802, this study) in an effort to benefit *M. peltatus* germplasm collection, conservation and future breeding.

In this study, *M. peltatus* was sampled from Baoting county in Hainan province of China (109.70° E, 18.63° N). A voucher specimen (Wang et al., GPSII-001) and its DNA was deposited in the Herbarium of the Institute of Tropical Agriculture and Forestry (code of herbarium: HUTB), Hainan University, Haikou, China.

The experiment is performed as reported in Zhu et al. (2018). Around six Gb clean data were assembled against the plastome of *Ricinus communis* (JF937588.1) (Rivarola et al. 2011) using MITO bim v1.8 (Le-Petit-Quevilly, France) (Hahn et al. 2013). The plastome was annotated using Geneious R8.0.2 (Biomatters Ltd., Auckland, New Zealand) against the plastome of *R. communis* (JF937588.1). The annotation was corrected with DOGMA (Wyman et al. 2004).

The plastome of *M. peltatus i*s found to possess a total length 163,304 bp with the typical quadripartite structure of angiosperms, contains two Inverted Repeats (IRs) of 27,112 bp, a Large Single-Copy (LSC) region of 89,886 bp and a Small Single-Copy (SSC) region of 18,840 bp. The plastozme contains 131 genes, consisting of 78 unique protein-coding genes (seven of which are duplicated in the IR), 30 unique tRNA genes (seven of which are duplicated in the IR) and four unique rRNA genes (5S rRNA, 4.5S rRNA, 23S rRNA and 16S rRNA). Among these genes, there are eight pseudogenes. The overall A/T content in the plastome of *M. peltatus* is 64.02%, which the corresponding value of the LSC, SSC and IR region were 66.30%, 70.40% and 57.80%, respectively.

**Figure 1.**
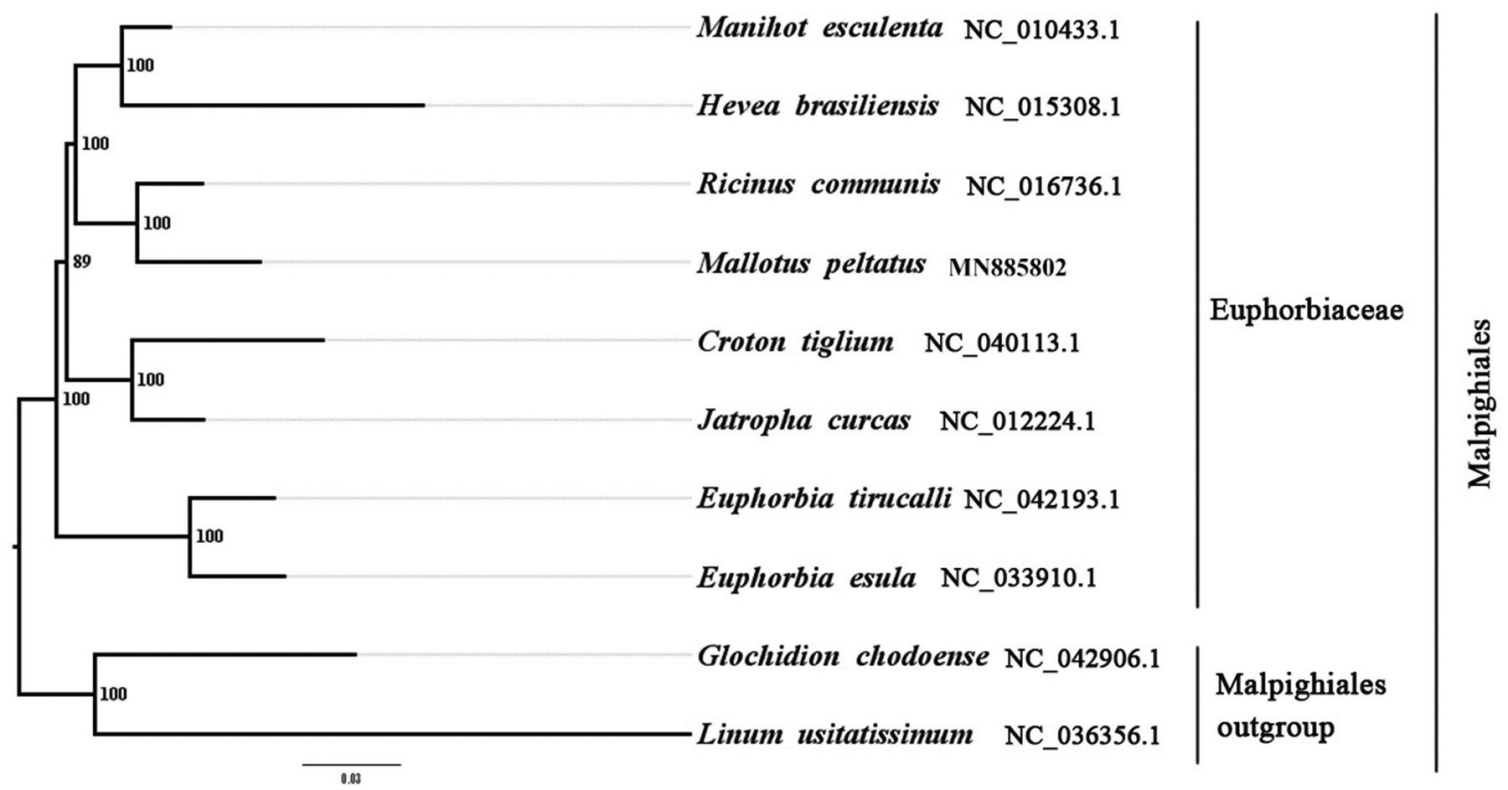
The best ML phylogeny recovered from 10 complete plastome sequences by RAxML. Accession numbers: *Mallotus peltatus* (GenBank accession number, MN885802, this study), *Manihot esculenta* NC_010433.1, *Hevea brasiliensis* NC_015308.1, *Ricinus communis* NC_016736.1, *Croton tiglium* NC_040113.1, *Jatropha curcas* NC_012224.1, *Euphorbia tirucalli* NC_042193.1, *Euphorbia esula* NC_033910.1, *Glochidion chodoense* NC_042906.1, *Linum usitatissimum* NC_036356.1.

We used RAxML (Stamatakis 2006) with 1,000 bootstraps under the GTRGAMMAI substitution model to reconstruct a maximum likelihood (ML) phylogeny of seven published complete plastomes of Euphorbiaceae, using *Glochidion chodoense* (Phyllanthaceae, Malpighiales) and *Linum usitatissimum* (Linaceae, Malpighiales) as outgroups. The phylogenetic analysis indicates that *M. peltatus* is closer to *R. communis* than other species in this study (Figure 1). Most nodes in the plastome ML trees were highly supported. With the complete plastome sequence of *M. peltatus* plastome , its resource exploitation and conservation project can be better proceeded, and phylogenetic studies of Euphorbiaceae can be explored more sufficiently.
